# Does female control and male mating system predict courtship investment and mating outcomes? A comparative study in five widow spider species (genus *Latrodectus*) tested under similar laboratory conditions

**DOI:** 10.1186/s12862-024-02272-9

**Published:** 2024-06-27

**Authors:** Luciana Baruffaldi, Maydianne C. B. Andrade

**Affiliations:** 1 Departments of Biological Sciences and Ecology & Evolutionary Biology, University of Toronto Scarborough, Toronto, ON M1C 1A4 Canada; 2 Departamento de Ecología y Biología Evolutiva, Instituto de Investigaciones Biológicas Clemente Estable, Montevideo, Uruguay

**Keywords:** Female control, Male courtship investment, Interspecific study, Sexual conflict

## Abstract

**Background:**

Male courtship investment may evolve in response to the male’s expectation of future mating opportunities or the degree of female control during mating interactions. We used a comparative approach to test this hypotheses by assessing the courtship and mating behaviors of five widow spider species (genus *Latrodectus*) under common laboratory conditions. We predicted male investment in courtship would be higher in species where males mate only once because of high cannibalism rates (monogyny, *L. geometricus, L. hasselti, L. mirabilis*), compared to species with rare cannibalism (*L. mactans, L. hesperus*) in which males should reserve energy for future mating opportunities. Increased male investment, measured as courtship duration, might also evolve with increased female control over mating outcomes if females prefer longer courtships. We tested this by assessing the frequency of copulations, timing of sexual cannibalism, and the degree of female-biased size dimorphism, which is expected to be negatively correlated with the energetic cost of rebuffing male mating attempts.

**Results:**

Copulation frequency was consistently lower in species with extreme female-skewed size dimorphism, and where sexual cannibalism was more prevalent, suggesting the importance of female control for mating outcomes. We confirmed significant interspecific variation in average courtship duration, but contrary to predictions, it was not predicted by male mating system, and there was no consistent link between courtship duration and sexual size dimorphism.

**Conclusion:**

We show that the degree of sexual dimorphism is not only correlated with sexual cannibalism, but also with mating success since restriction of male copulation frequency by female *Latrodectus* affects paternity. However, predictions about male mating system or female control affecting courtship duration were not supported. We propose that the form of female control over mating and cannibalism, and male responses, might be more informative for understanding the evolution of courtship duration. For example, male tactics to avoid female aggression may drive lower courtship duration in species like *L. mirabilis*. Nonetheless, our results differ from inferences based on published studies of each species in isolation, illuminating the need for standardized data collection for behavioural comparative studies.

**Supplementary Information:**

The online version contains supplementary material available at 10.1186/s12862-024-02272-9.

## Background

Despite being costly for males, persuasive courtship precedes mating in many taxa [1, 2]. Courting males incur energetic costs and lost opportunity costs, since time invested in one potential mate requires forgoing other activities (e.g. foraging, mating with other females, [1–3]). Courtship is expected to evolve and be maintained when females influence mating outcomes, and female receptivity is linked to courtship [4, 5]. Given the trade-offs for males, investment in courtship is predicted to vary as a function of a variety of factors, including the female’s preferences for long or short courtships, the male’s energetic reserves and size, the risk of being usurped prior to mating, the potential reproductive value of the female being courted, and the male’s expectation of future mating opportunities [6–10]. Courtship duration is one important, but relatively understudied, aspect of this trade-off. Variation in time spent courting is important since the male’s energetic costs, time costs, and risk of competitor interference will increase with courtship duration, but for choosy females, longer courtship may provide more information and time for male assessment (although courtship may also increase exposure or vulnerability to predators) [9, 11–14]. Theory suggests that males will thus attempt to minimize courtship duration [15] in anyone mating, where this will be more evident in polygamous males that may allocate effort across multiple mating attempts. However, if females prefer longer courtships, this effect may be counteracted, generating sexual conflict over courtship duration [1–4]. Under sexual conflict, the average evolutionary outcome should be shifted towards the female’s fitness interests in species with higher degrees of female control over mating outcomes. For example, in species with female-biased sexual size dimorphism, females can avoid or neutralize early copulation attempts by males and enforce longer courtship with relatively little cost [16]. However, in species where females and males are more similar in size, even if there is a female preference for longer courtship, constantly removing or discouraging persistent male mating attempts may be sufficiently energetically costly that females may reduce their resistance (e.g., [17]). Thus, lower size dimorphism may be linked to decreased courtship duration via decreases in the likelihood of females expending time or energy to rebuff persistent mating attempts, or decreased effectiveness of such rejection behaviours.

One approach to testing this idea within species is to determine whether courtship duration is negatively correlated with the expected value of future matings for males (= residual reproductive value, [18]), and positively correlated with the degree of female control. However, since both of these variables may shift across mating pairs due to factors such as age or size (e.g., [19]), an intraspecific approach can be challenging because it requires deciphering the effects of both male and female decisions on courtship outcomes [20–22]. This hypothesis can also be tested using comparative approaches since it predicts that interspecific differences in average courtship duration should evolve in concert with variables predicting the outcome of sexual conflict across species. Although contextual dynamics will still be important, comparative analyses can suggest whether overarching correlations are consistent with the sexual conflict hypothesis [23].

Comparative analyses often involve mining the literature for data to allow broad comparisons [24]. However, this approach can be problematic, particularly for behaviour. Behavioural data collected in different laboratories and contexts can be variable, even after using standardized techniques [25], due to a variety of factors related to variation among study animals and experimenters [26–28], as well as variation in data coding [29], and environmental factors such as testing temperatures (e.g., [30, 31]). To reduce errors in inference from confounding variables, it would be ideal to collect behavioural data from multiple species reared and tested under similar laboratory conditions. However, this necessarily limits the scale of such investigations, and can create a tension between designs that maximize the number of species sampled versus those that prioritize the reliability of the comparative data. It is useful, therefore, to compare inferences from the literature with those arising from data collected using standardized methods.

Here we collected data to allow an initial examination of broad predictions of the sexual conflict hypothesis for the evolution of courtship duration, using congeners of *Latrodectus* spiders with species-level differences in male residual reproductive value, and variables related to female control such as the frequency of sexual cannibalism and the degree of sexual size dimorphism ([32, 33], A secondary goal of this study was to assess whether results derived when all species were tested under common conditions are consistent with inferences from the existing literature. We reared unmated males and females of five species of *Latrodectus* spiders (*L. mirabilis, L. hasselti, L. geometricus, L. mactans, L. hesperus*) from field-collected egg sacs under similar laboratory conditions (e.g. temperature, diet), measured the degree of sexual size dimorphism of adults, and then conducted conspecific mating trails. We assessed courtship duration and indicators of female control over mating attempts and male mating success (mating frequency, number of copulations and occurrence and timing of sexual cannibalism).

The *Latrodectus* clade is appropriate for testing the sexual conflict hypothesis for courtship duration because the literature across the genus suggests interspecific variation in the degree of sexual dimorphism, frequency of sexual cannibalism, average courtship duration (a tenfold difference), and male mating system (number of potential mates encountered in a lifetime) [34–36]. There are three key aspects of *Latrodectus* behaviour and biology that are relevant to its utility for this study. First, there are several indicators that *Latrodectus* females prefer prolonged courtship periods from males. A common observation during mating interactions is the repeated approach of courting males to females [14, 37], with cycles of mounting (or mounting attempts) rebuffed by the female when they strike at or push the male with their legs. Some females eventually adopt a receptive posture [38, 39], but females may also flick males off the web entirely [36, 37]. Moreover, while male courtship begins as somewhat chaotic movements creating low-amplitude impulses that allow differentiation from prey [39], over time it progresses into a structured sequence of higher-amplitude signals with distinct components that signal male traits [14, 40]. Attenuating components of the signal leads to delays in female receptivity and increased latency to copulation, perhaps because females are insufficiently stimulated [14]. In unmanipulated pairings, males that attempt to mate relatively rapidly are killed by females before a complete mating is achieved [41]. For the focal species in this study, the literature reports courtship durations that range from ~ 20 min in *L. mirabilis* [42], ~ 120 min in *L. hesperus* [43], ~ 200 min in *L. geometricus* [44], and ~ 300 min in *L. hasselti* [45]. However, these measurements of courtship duration arise from different studies under variable conditions (e.g. temperature, diet, developmental history, lab reared females versus field collected) thus it is unclear whether these results represent consistent species-level divergence in courtship duration and behaviour.

Second, although female-biased size dimorphism is a feature of all *Latrodectus*, the degree of dimorphism apparently varies across congeners (e.g., [35], although measurement of inter-specific differences using common-garden rearing are rare). Moreover, within species, the likelihood of sexual cannibalism increases as the size ratio of female to male increases (e.g., in *Latrodectus tredecimguttatus*, [33], consistent with the literature on spiders in general [46], and other web-building spiders that show female-biased size dimorphism in particular [32]. The proposed explanation is that females decide to exert physical control as a function of the degree of size dimorphism [33], consistent with the idea that there is a higher energetic and time cost of rebuffing relatively larger compared to relatively smaller males. For example, although body mass may differ by a factor of 100 for some *Latrodectus*, the degree of sexual size dimorphism in leg length is much smaller in some species (e.g., [34, 35, 43]. Relatively long-legged males may be better able to clasp the female’s abdomen and thus resist female attempts to push them off during mounting, increasing the energetic cost of rejection by females.

**Fig. 1:**
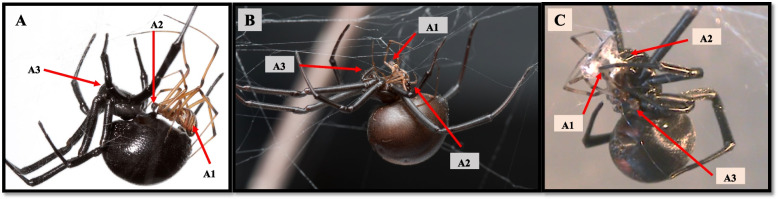
Pictures of female and male from different species of *Latrodectus* in copula representing the different mating copulatory positions and behaviours observed in females and males (A1 = male abdomen, A2 = male insertion of copulatory organ, A3 = female fangs). Picture **A** shows *Latrodectus hesperus* copulating in the posture also observed in *L. mactans*, where the males rest his abdomen on top of the female’s abdomen during mating. Picture **B** shows *L. hasselti *copulating in the posture also adopted by *L. geometricus,* where the male is in the ‘somersault’ position, placing their abdomen in close proximity to the fangs of the female (A1 = male abdomen, A2 = male insertion, A3 = female fangs). Picture **C** shows *L. mirabilis* in copula with the male wrapped in silk by the female, and the female clasping the male’s abdomen near to her fangs. Pictures A and B were taken by Sean McCann, picture C was taken by LB

Third, interspecific variation in the male mating system in *Latrodectus* is predicted to lead to variation across species in the payoff for high male investment in one mating opportunity. *Latrodectus* males have finite energetic reserves to invest in mate searching and courtship since they do not eat as adults [47], and mortality during mate searching is high ([48], and see review in [36]). Moreover, while males are able to mate repeatedly in some species (polygyny), in others, females cannibalize or injure males during mating attempts so frequently that a single mating opportunity is common (i.e., monogyny [46, 49]). In monogynous species, selection favours ‘terminal investment’, through which investment in mating occurs without males reserving resources for (rare) future mating opportunities [50]. In the focal species in our study, varied male mating systems predict different levels of investment in a given mating, and this is reflected in interspecific differences in copulatory behaviour and outcomes. For example, the initial copulatory position is very similar across the genus, with the male mounting the female, venter to venter, with the same anterior-posterior orientation, and his abdomen far from the females’ fangs (Fig. 1A). Male *L. hesperus* and *L. mactans* remain in this position during mating, (Fig. 1A), and in general they are not attacked during copulation (only when the females are hungry e.g., *L. hesperus* [35, 51]; *L. mactans* [36, 52]), and males are considered polygynous and relatively long-lived [36, 52]. In contrast, relatively short-lived male *L. hasselti* [45, 53], *L. geometricus* [38], and *L. mirabilis* [42] are typically monogynous. During copulation in *L. hasselti* and *L. geometricus,* most males twist their bodies above the female’s fangs (somersault behaviour) and are cannibalized during sperm transfer [36] (Fig. 1B). In *L. mirabilis* sexual cannibalism also occurs at a high rate, beginning when females clasp and pierce the male’s legs during copulation, then proceeding when females pull the male’s abdomen onto their fangs and terminate the copulation [42] (Fig. 1C). Thus, males of these three species are considered to be monogynous, although this is initiated by male behaviour in *L. geometricus* and *L. hasselti*, but by female behaviour in *L. mirabilis.* This difference in mating system is also correlated with male mate choice. Whereas *L. hasselti* males do not discriminate hungry females and court them regardless of the risk of cannibalism [54], polygynous *L. hesperus* males can detect poorly fed (hungry) females at a distance, avoid them, and are unlikely to court them [51, 54, 55].

In spiders, links between courtship and sexual cannibalism often focus on courtship as a means of reducing the female’s cannibalistic tendencies, and often predict longer, more cautious courtship will evolve when the risk of pre-copulatory cannibalism is high [46, 49]. Here we tested sexual conflict hypotheses for variation in courtship and mating by focusing on male investment decisions and the effect of the degree of sexual dimorphism, and therefore female control, on mating outcomes. First, we predicted that in *Latrodectus* species in which males have a low expectation of future mating (*L. mirabilis, L. hasselti, L. geometricus*), longer average courtship durations would evolve. In comparison, in polygynous congeners males may allocate effort across multiple mating opportunities and so invest less in courtship with any one female (*L.hesperus, L. mactans*). Second, assuming that more extreme female-biased sexual size dimorphism reduces the energetic cost of rebuffing males, we predicted longer courtship on average in species with more extreme sexual size dimorphism. We also expected that species with more extreme dimorphism would show and increased likelihood of females physically controlling mating outcomes, manifested as fewer copulations and elevated frequencies of sexual cannibalism [9, 36, 41].

## Results

### Sexual size dimorphism varies under common rearing conditions

**Fig. 2:**
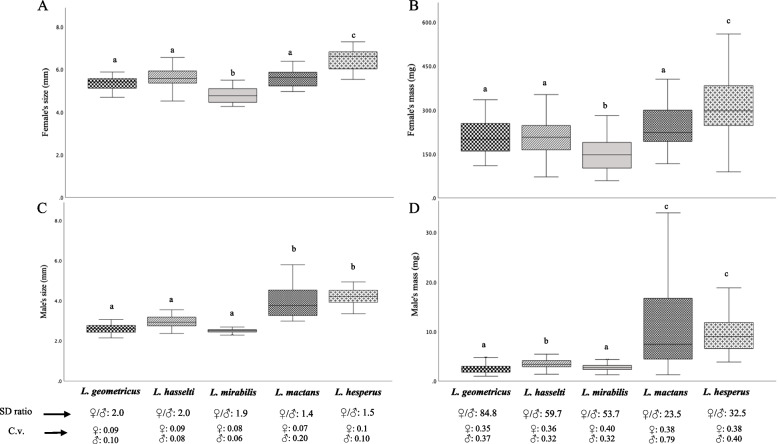
Boxplots of female body mass/size (**A**, **B**) and male body mass/size (**C**, **D**) for the five *Latrodectus* species along with the sexual size dimorphism ratio (female/male mass/size) and coefficient of variation for mass/size in each of the five species. Size was measured as the average combined length of the patella and tibia of the front two legs. Boxes with different superscripted letters (a, b, c) indicate significant differences at *p* ≤ 0.05 in post-hoc pair-wise tests. Boxplots show medians (centre line), range between the first and third quartiles (box), and the maximum and minimum values (whiskers)

Female size and mass showed significant variation among species (size: One-way ANOVA: *F* = 28.66, *p* < 0.001; mass: Kruskal-Wallis: H = 56.72, *p* < 0.001) with *L. mirabilis* females having the smallest size/lowest mass, and *L. hesperus* having the largest size/highest mass of all 5 species (Fig. 2A and B). Variation in size within each female species was similar (coefficient of variation, CV range = 0.1 - 0.07), and the same was observed with female mass (CV range = 0.40 - 0.35, Fig. 2).

Male size also showed significant variation among species (*F* = 81.12, *p* < 0.001) with *L. geometricus*, *L. hasselti* and *L. mirabilis* similar in size, and significantly smaller than males of *L. mactans* and *L. hesperus* (Fig. 2C). Similar results were observed with male mass (*H* = 202, *p* < 0.001), with males of *L. mactans* and *L. hesperus* the heaviest of all the species (Fig. 2D). Variation in size/mass within males of each species was similar (CV range size/mass = 0.1-0.06/ 0.32-0.4) with the exception of *L. mactans* males, in which variation in size/mass was roughly two times higher than other species and conspecific females despite common rearing conditions (CV size/mass = 0.20/ 0.79, Fig. 2).

All species showed female-biased size/mass dimorphism (Fig. 2). Average mass differed by an order of magnitude between males and females of each species, and the difference was particularly extreme for *L. geometricus, L. mirabilis* and *L. hasselti* (females more than 50 × heavier than males). However, leg length dimorphism was less extreme, particularly for *L. hesperus* and *L. mactans* in which male’s legs were approximately ¾ the length of female’s legs, in comparison to *L. geometricus*, *L. mirabilis* and *L. hasselti* for which male’s legs were ½ the size of female’s legs.

### Mating trials

#### Comparison of mating outcomes and copulation frequency

In all species, most males courted females actively from the time they were first in contact with the web (≥ 75% of trials), and most males that courted also mounted females (≥ 92% of courting males, Table 1) with no differences among species (courtship: χ^2^ = 5.48, *p* = 0.24; mounting: χ^2^ = 1.92, *p* = 0.75). Despite similar mounting frequency, mating success of *L. geometricus* was lower (40% of males that mounted, Table 1) compared to the other species (χ^2^ = 16.92, *p* = 0.002), which all showed similar, high mating success (≥ 83% of males that mounted also mated, χ^2^ = 0.69, *p* = 0.87).

**Table 1:** Overview of the outcome of laboratory trials in which we show the frequency of males that proceeded from one level of mating interaction to the next level of mating interaction until achieving copulation for the five species of *Latrodectus* spiders

Mating outcome/ species	Male courtship	Male mounting	Mating	Two copulations	Male somersault	Pre-copulatory cannibalism	Copulatory cannibalism
*Latrodectus geometricus* (N = 23)	21 (91%)^a^	20 (95%)^a^	8 (40%)^a^	4 (50%)^a^	7 (88%)^a^	0^a^	4(50%)^a^
*L. mirabilis* (N = 15)	13 (87%)^a^	12 (92%)^a^	10 (83%)^b^	6 (60%)^a^	0^b^	0^a^	3 (30%)^a^
*L. hasselti* (N = 15)	15 (100%)^a^	15 (100%)^a^	13 (86%)^b^	3 (23%)^a^	13 (100%)^a^	0^a^	11* (92%)^b^
*L. mactans* (N = 16)	12 (75%)^a^	12 (100%)^a^	10 (83%)^b^	9 (90%)^b^	0^b^	0^a^	0^c^
*L. hesperus* (N = 19)	15 (79%)^a^	14 (93%)^a^	13 (93%)^b^	12 (92%)^b^	0^b^	2 (11%)^b^	0^c^

^a,b,c^Within each column, different superscripts indicate values that are significantly different from each other at *p* < 0.05 (see text for details). *Data missing from 1 trail

In this genus females have two sperm storage organs and first-male sperm precedence within each organ, so if females permit a male to copulate twice, that male will father most of the female’s offspring [56]. There was significant variation in the frequency with which males achieved two copulations (χ^2^ = 17.42, *p* = 0.0016). For the two polygynous species, *L mactans* and *L. hesperus*, almost all of the males that mated achieved two copulations (90% and 92% respectively) whereas only about half of males copulated twice in *L. mirabilis* and *L. geometricus*, and very few *L. hasselti* males copulated twice (23% of males that mated). The low frequency of paired copulations in *L. hasselti* arises mainly from a very high frequency of sexual cannibalism during the first copulation (69%, ‘premature cannibalism’). In contrast, the rates of “premature cannibalism” were very low in *L. geometricus* (12.5%) and in *L. mirabilis* (10%) compared to *L. hasselti* (χ^2^ = 11.15, *p* = 0.0038). The likelihood of cannibalism across the entire mating was 50% for *L. geometricus* and 30% for *L. mirabilis* compared to 92% for *L. hasselti* (χ^2^ = 7.21, *p* = 0.027). For *L. hesperus*, cannibalism occurred rarely, and only during the initial courtship, preventing mating (11%), and there were no occurrences of cannibalism in *L. mactans* (Table 1).

#### Comparison of courtship and mating behaviours

**Fig. 3:**
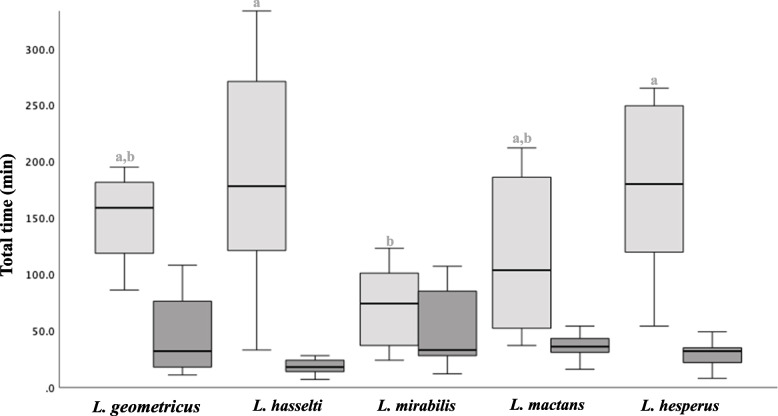
Box plots comparing duration of: (1) total male courtship behaviour ( from the beginning of the trial until achieving the first copulation, in light grey), and (2) total mating (from the beginning of the first copulation until the end of the second copulation in dark grey). Different letters (a) above the boxes indicate significant differences (*p* ≤ 0.05) among the species within each data type based on Tukey’s post-hoc tests (Supplementary material). Boxplots show medians (centre line), range between the first and third quartiles (box), and the maximum and minimum values (whiskers)

**Fig. 4:**
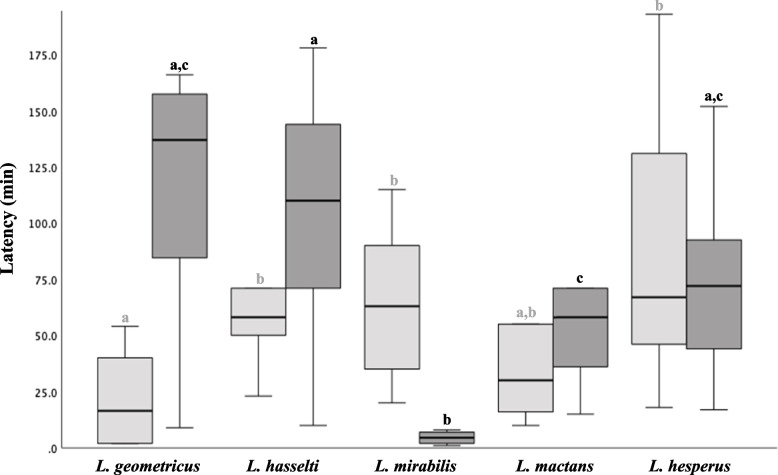
Box plot comparing male courtship latency for all the study species. Courtship is divided into: (1) pre-mounting courtship (distal courtship, before mounting the female’s ventral abdomen close to the epigine, in light grey), and (2) post-mounting courtship (proximal courtship, after mounting the female’s venter and before achieving the first copulation, in dark grey). Different letters above columns indicate significant differences (*p* ≤ 0.05) among species based on Mann Whitney’s post-hoc tests (Supplementary material). Boxplots show medians (centre line), range between the first and third quartiles (box), and the maximum and minimum values (whiskers)

Total courtship duration (from the beginning of the trial until the first copulation) differed significantly among the species tested here (One way ANOVA *F* = 4.26, *p* = 0.005, Fig. 3). *L. mirabilis* males courted for just over one hour (73.1 ± 36.2 min), which was the briefest courtship among species, although there was some overlap with courtship durations of *L. mactans* and *L. geometricus* (Fig. 3). *L. hasselti* and *L. hesperus* were very similar (Tukey’s post-hoc comparison, *p* = 0.97), with courtship durations almost twice as long as *L. mirabilis* (~ 150 min, Fig. 3). The time invested in courtship components prior to the first mount (pre-mounting courtship, Kruskal-Wallis *H*, *p* = 0.007), and between mounting and copulation (post-mounting courtship; *H* = 24.5, *p* < 0.001) differed more widely among the five species (Fig. 4). *L. geometricus* males mounted females faster than other species, less than 30 min after the commencement of trials. *L. mactans* were intermediate, and *L. hasselti*, *L. mirabilis*, and *L. hesperus* were all longer and similar, courting for almost 60 min before mounting females (*H* = 0.64, *p* = 0.72). Although *L. geometricus* males mounted rapidly, they exhibited the highest number of alternating cycles of courting on the web and female’s venter before copulation (62.1 ± 46.9 SD abdominal mounts). At the opposite extreme, once *L. mirabilis* mounted females, they rarely dismounted prior to copulation (1.1 ± 0.32 abdominal mounts, *H* = 25.7, *p* < 0.001, and see [32]). *L. hasselti* (10.8 ± 10.17)*, L. mactans* (22.7 ± 11.38)*,* and *L. hesperus* (22.5 ± 15.92) had an intermediate number of cycles of mounting and dismounting the females’ abdomen before mating (*F* = 3.22, *p* = 0.055).

*L. mirabilis* males also achieved the first copulation significantly more rapidly after mounting the female (post-mounting courtship, 10.6 min ± 20 min SD, Fig. 4) than males of the other species, which typically took 5 × longer in post mounting courtship before copulation was achieved (*F* = 2.24, *p* = 0.10). As suggested by the literature, every *L. hasselti* male somersaulted, and *L. geometricus* was the only other species in which somersaulting occurred, although at a lower rate (88%, Table 1)*.* The duration of the first copulation also varied (*F* = 9.42, *p* < 0.001), with *L. mirabilis* males copulating for the longest (31 ± 17.9 min), and *L. hesperus* the shortest (7.75 ± 2.38 min) periods. *L. hasselti* (16.1 ± 6.33 min)*, L. geometricus* (20.3 min ± 8.46), and *L. mactans* (12.4 ± 4.88 min) had similar, intermediate copulation durations (*F* = 3.19, *p* = 0.056). However, the total mating duration (from the beginning of the first copulation until the end of the last copulation) was not different among species (*H* = 6.54, *p* = 0.16, Fig. 4).

## Discussion

In our study we found sexual dimorphism was related to copulation frequency, with less dimorphic species achieving two copulations in most matings (*L. mactans*, 90% of trials; *L. hesperus*, 92% of trials), whereas in the three more dimorphic species (*L hasselti, L geometricus, L. mirabilis,* Fig. 2), double-copulation matings were much less common (from 60% to only 23% of matings, Table 1), despite repeated attempts by surviving males. As has been showed previously in interspecific and intraspecific studies of other spider taxa [16, 32, 57], including *Latrodectus tredecimguttatus* [33], sexual cannibalism was most common in those species in which there was more extreme female-biased size dimorphism. Although all *Latrodectus* spiders show extreme female-biased dimorphism with respect to mass, dimorphism is less extreme for leg lengths, particularly in *L. hesperus* and *L. mactans* (Fig. 2). Thus, consistent with our predictions, females may be less likely to attempt to physically manipulate or cannibalize males that are relatively large (species with less extreme female-biased sexual size dimorphism [16, 57]), perhaps as an evolutionary response to variation in the cost of rebuffing males that vary in their ability to resist [32, 33, 57]. We infer that a similar effect may shape copulation frequency, suggesting a role for female control, but manifesting primarily via sexual cannibalism and species-specific variation in copulation frequency, and thus the likelihood of first male sperm precedence (e.g., see [36]). Lower copulation frequency may be costly for males as it reduces the likelihood of first male sperm precedence [56] if the female mates again, but it may be beneficial for females as they then retain the capacity for post-copulatory choosiness [58]. A broader comparative analysis of copulation frequency, sexual size dimorphism and remating behaviour across spider genera in which cannibalism has been reported would be required to test this hypothesis.

We focused here on sexual conflict hypotheses for explaining the evolution of variation in courtship duration among species, focusing on *Latrodectus* species as model clade, predicting higher investment in longer courtship in species where females have stronger control over mating, and where males have lower expectation of future reproduction [2, 4]. The courtship durations reported previously in the literature anecdotally suggested support for these predictions. For example, published estimates of courtship in the monogynous *L. hasselti* centre around 300 min [45], more than double the courtship period reported for polygynous *L. hesperus* (~ 120 min [43, 59]). Under standardized conditions we found species-specific differences in courtship duration, but no consistent inter-specific relationship between courtship duration, or the duration of common courtship components, and male expectation of future mating (Figs. 3 and 4). Instead, courtship durations in the polygynous species *L. hesperus* and *L. mactans* were similar to those in the monogynous *L. hasselti* and *L. geometricus*, and the shortest courtship overall was for monogynous *L. mirabilis* (Fig. 3). Although it is common to use data gleaned from a variety of sources in the literature to support comparative analyses of behaviour, our work thus suggests a caution to this approach. We also found no support for the second prediction, that courtship duration would be longer if female control over mating outcomes was higher (estimated by the degree of female-biased size dimorphism, e.g., [57]). Here again, we found no consistent pattern. The longest proximal (pre-mounting) courtship periods were found in two of the highly dimorphic species (*L. hasselti*, female/male size ratio = 2.0; *L. mirabilis*, size ratio = 1.9), but also in a species with significantly lower dimorphism, (*L. hesperus*, female/male size ratio = 1.5, Fig. 2). Our results do suggest a role for female control but manifesting primarily via sexual cannibalism and species-specific variation in copulation frequency, and thus the likelihood of first male sperm precedence (e.g., see [36]).

Our results suggest an alternative link between courtship and sexual cannibalism [46, 57]. We propose that the brief courtship that has evolved in a species like *L. mirabilis* may primarily reflect male attempts to rapidly mount females to avoid cannibalistic attacks (e.g., as proposed for orb-weaving spiders by [8, 60, 61]). In the species studied here, longer total courtship was observed in species where there is no (*L. mactans*) or low rates of cannibalism (*L. hesperus*), or where cannibalism only occurs as part of a male mating strategy during copulation (*L. hasselti* and* L geometricus*, Fig. 3). For these species, male courtship investment included time-consuming alternating cycles of movement on and off the female’s abdomen which occurred repeatedly in *L. hasselti* (~ 10 cycles), as well as *L. hesperus* and *L. mactans* (~ 23 cycles in each species). Even in *L. geometricus*, in which males’ initial mounting of females occurred more rapidly than any other species (Fig. 4), courtship continued for long periods, with males moving on and off the female’s abdomen an impressive 62 times on average before copulation. However, in *L. mirabilis*, in which females initiate copulatory cannibalism at high rates [42], males presented the shortest total courtship and males appear to scramble for a relatively safe perch on the female’s abdomen, then attempt copulation relatively quickly, rarely returning to the web after the initial mount ([42], Fig. 4). Comparing the results reported here to an earlier study also suggest indirect support for this idea. Even though males of *L. mirabilis* had the briefest courtship of all the species in this study (~ 70 min), it was more than double the courtship duration reported in a previous study of *L. mirabilis* (~ 20 min [42]) where males were paired with females collected as subadults from the field. These field-collected females were much more cannibalistic (70% of the trials [42]) than those reared through a generation in the lab for this paper (30%). This could indicate that males of *L. mirabilis* assess cannibalistic tendencies in females (perhaps by sex pheromones as reported for males of *L. hesperus* [51, 54, 55]) and use that information to adjust their courtship and the timing of seeking a safe perch on female’s abdomen. Alternatively, as we propose, this may be an evolved shift in male behaviour in this species, arising from a history of sexual conflict.

The drop in the rate of sexual cannibalism for lab-reared females of *L. mirabilis* relative to previous a previous study [42] may be mediated by generous laboratory diets, since cannibalism is more likely when females are hungry in some species (e.g., *L. hesperus* [51]*,*), or when food intake is unpredictable or low during development (e.g., *N. plumipes* [62]). However, not every species showed a change in cannibalism rates. For example, although there is evidence that body condition of *L. hasselti* females affects cannibalism in nature, the occurrence of cannibalism for lab-reared females here (92%) was higher than reported from unmanipulated matings in nature (65%, [63]). As an alternative, these effects in *L. hasselti* could be related to female perception of male availability in the lab compared to the field, rather than diet, since females employ cannibalism as a mechanism of cryptic choice [58]. In our mating experiments, trials were run in open arenas held in a common room with other conspecific pairs, allowing the detection of airborne pheromones indicating the proximity of other potential mates. There is much more to understand here, but these results suggest the importance of considering the extent to which female, as well as male, behaviour can be affected by laboratory rearing and experimental conditions, particularly when testing comparative hypotheses.

## Conclusion

Overall, this study suggests neither variation in future mating opportunities of males nor the degree of female control over mating have a strong influence on male courtship investment. However, female control during copulation, measured as the degree of sexual dimorphism, may allow species with relatively large females to restrict male copulation frequency compared to species with less extreme sexual dimorphism, for which mating outcomes may more closely match the male optimum. Additional data from this genus would allow a broader comparative test of this hypothesis, and also allow exploration of the idea that female control is key to understanding mating dynamics (see Fig. 4 in [42], for an illustration of gaps in information in the genus). Nevertheless, these results make it clear that data mining approaches may not be sufficient, and standardizing conditions of data collection are important for a robust analysis.

## Methods

### *Latrodectus* species

All five focal spiders were from outbred laboratory populations established from mated females collected in the field (*L. hasselti*: Sydney, Australia; *L. mirabilis* Canelones, Uruguay; *L. geometricus*, Florida, USA; *L. mactans*, Oklahoma USA; *L. hesperus*: California, USA), representing the two described *Latrodectus* clades and different biogeographic regions: *L. geometricus (geometricus clade), L. hasselti, L. hesperus*, *L. mactans* and *L. mirabilis,* (mactans clade, see [64]). Spiders were held in individual clear plastic cages (5 × 5 x 7 cm, Amac Plastics, Ltd.) in temperature-controlled rooms at ~ 25° C (with occasional variation from 23 to 29 °C) on a 12:12 h light cycle. Juveniles and males of all species were fed twice weekly with *Drosophila sp*. fruit flies and females were fed one subadult cricket (*Gryllodes sigillatus*) once per week after the 5th instar (when females become much larger than males). Spiders were monitored weekly in order to record the date of their final moult (after which they are sexually mature adults).

In *Latrodectus* spiders, males begin to court when they come in contact with pheromones on the female’s silk, and courtship involves vibratory signals on the female’s web that become more structured as the courtship progresses [14, 36, 39]. Eventually males mount the female abdomen, and in many species males alternate intervals of courtship on the female’s abdomen with intervals when they return to and signal on the web [37] until attempting copulation.

Copulation occurs when the male climbs onto the female’s abdomen with their ventral surfaces in close proximity, their cephalothoraxes facing in the same direction and male inserts one of his copulatory organs inside the female genital openings. Female widows’ external genitalia include two separate genital openings, each of which is connected to one of two sperm storage organs (spermathecae) from which sperm are taken in roughly equal proportions at fertilization [56, 65]. Males have two copulatory organs (pedipalps), and if they copulate only once, one spermatheca will be available for insemination by rivals that females may mate with later, which will reduce the paternity of the first mate [56]. Thus, males must inseminate both spermathecae to ensure their paternity, and this is accomplished only if they copulate twice, inserting one of their two pedipalps at each copulation [56].

### Sexual dimorphism: body mass and size

For laboratory reared spiders, body mass and size are correlated [66]. We assessed sexual size dimorphism based on mass and size for spiders reared under the same conditions for a sample of lab-reared spiders. All spiders (N for females, males: *L. geometricus* = 45, 68; *L. hasselti* = 42, 72; *L. mirabilis* = 46, 71; *L. mactans* = 23, 22; and *L. hesperus* = 42, 78) were weighed using an analytical balance (Ohaus electronic balance, accurate to 0.01 mg). For the estimation of body size, spiders (N for females, males: *L. geometricus* = 20, 22; *L. hasselti* = 21, 20; *L. mirabilis* = 20, 21; *L. mactans* = 17, 16; and *L. hesperus* = 22, 26) were photographed using a dissecting microscope (Zeiss Stemi 2000-C) and a digital camera (Nikon DXM 1200). Photos were taken of the female’s and male’s patella-tibia length in both left and right front legs, which provides an accurate representation of body size in spiders [47]. The photos were later analyzed and measured using Image J. Sexual dimorphism was calculated as female mass/ male mass, and female size/male size independently. We also report the coefficient of variation for mass and size (CV = standard deviation/mean). This measure of variation can be meaningfully compared across groups with different mean sizes.

### Mating trials

Our mating trials (Table 1) were run in blocks in one room, with several open arenas of conspecifics run simultaneously to ensure spiders could detect airborne pheromones of conspecifics, which may affect behavioural decisions related to the expectation of future mating opportunities [55, 58, 67].

Once females and males reached sexual maturity, they were randomly paired with unrelated conspecifics for mating trials. Females were placed in mating arenas for 48 h to construct webs prior to the introduction of males. Web supports consisted of two inverted U-shaped metal frames attached in parallel on a plastic support (11 × 8 × 8 cm) placed inside an experimental container (35 × 30 × 15 cm). Females readily build a snare and capture threads (viscid threads) similar to that seen in nature on these frames (also used in [37, 42, 68]). On trial days, males were introduced as far from the adult female as possible by allowing them to drop from a dragline onto the web. Trials continued from male introduction until 2 copulations were achieved, until the male was dead, or until at least 6 h had passed with no courtship or mating. All trials were recorded on digital video using Panasonic low-light black and white cameras (WV BP330) with macro zoom lenses (Navitar Macro-Zoom 7000) under low-lux red-light illumination.

Videos were later analyzed by one of us (LB). We described and quantified behaviours of males and females that reflect established stages of mating interactions, as well as the duration of the stages from male courtship on the web through to copulation [37, 42, 68]. We defined a successful mating as one in which males performed one or two palpal insertions (copulations) with visible haematodocha expansion (e.g., [69]). We assessed the proportion (frequency) of trials that (1) reached different mating levels in the progression of behaviours leading to and including copulation (in order: male courtship, male mounting, mating, one copulation, two copulations) and, (2) ended with pre-copulatory or copulatory sexual cannibalism. We quantified total courtship duration as total time from the start of the trials until the first copulation (males courted throughout this period). We also broke down the components of courtship in terms of how much time the male invested on: (1) pre-mounting courtship (or ‘distal courtship’, [59]): time from the start of the trial to the first mount (defined as when the male first moves onto the female’s ventral abdominal surface (venter), which is the location of the female’s genital opening), and (2) post-mounting courtship (or ‘proximal courtship’ [39, 40, 59]): time from the first mount until the first copulation. These are meaningful courtship components as there is a shift in the structure of the signals produced by *Latrodectus* males after mounting [14, 40]. Cycles of courtship alternating between the female’s web and venter are typical of *Latrodectus* [37] and may also reflect courtship investment, so we also quantified the number of these cycles by counting the number of times the males mounted the female’s venter prior to the first copulation. Copulation duration was quantified as the total time during which the male’s pedipalp was inserted in the female’s genitalia (clearly visible on videos and lasted 2 min or more), and total mating duration was quantified as total time since the beginning of the first pedipalp insertion (first copulation) until the end of the male’s final copulation.

### Statistics

#### Sexual size dimorphism

Female and male mass were not normally distributed (Shapiro-Wilk *p* < 0.05), therefore we performed the non-parametric global independent samples Kruskal-Wallis test and then pairwise comparisons among the different species (Mann-Whitney test). Female size and male size were normally distributed (Shapiro-Wilk *p* > 0.05), therefore we performed One-way ANOVA global test and then pairwise comparisons among the different species (Tukey’s Test).

#### Mating trials

We used χ^2^ tests to ask whether the proportion of trials that progressed to a particular mating level varied among species. We compared courtship, copulation, and total mating durations using One-way Anova and Tukey post-hoc tests if the data had a normal distribution, and Kruskal-Wallis and Mann-Whitney post-hoc test if the data did not have a normal distribution. Statistical analyses were conducted using The PAST package [70].

### Supplementary Information

Supplementary Material 1.

Supplementary Material 2.

## Data Availability

Supplementary material 1 includes the statistical results of pairwise comparisons and Supplementary material 2 includes all the raw data.
