# Supplementary material for: Does female control and male mating system predict courtship investment and mating outcomes? A comparative study in five widow spider species (genus Latrodectus) tested under similar laboratory conditions
Source: BMC Ecol Evol. 2024 Jun 27;24:86. doi: 10.1186/s12862-024-02272-9 (PMC11212240; doi:10.1186/s12862-024-02272-9)
Supplement: Supplementary file 1 — Supplementary Material 1. [file 12862_2024_2272_MOESM1_ESM.pdf]

Supplementary Material: Statistics analysis from the results sections of the paper.

1. Statistical results of body size and mass for the males and females of the species used in the paper.

Table 1. 1. Pairwise test results of male mass between the species used in the paper.

Species \ Species	Male mass (mg) Mann-Whitney pairwise				
	L.geometricus	*L. hasselti*	*L. mirabilis*	*L. mactans*	*L.hesperus*
L.geometricus	---------------	U = 1211, P < 0.001*	U = 2038, P = 0.066	U = 105.5, P < 0.001*	U = 14.5, P < 0.001*
L. hasselti	U = 1211, P < 0.001*	-----------	U = 1440.5, P < 0.001*	U = 222, P < 0.001*	U = 115, P < 0.001*
L. mirabilis	U = 2038, P = 0.066	U = 1440.5, P < 0.001*	------------	U = 125, P < 0.001*	U = 33, P < 0.001*
L. mactans	U = 105.5, P < 0.001*	U = 222, P < 0.001*	U = 125, P < 0.001*	-----------	U = 771.5, P = 0.42
L. hesperus	U = 14.5, P < 0.001*	U = 115, P < 0.001*	U = 33, P < 0.001*	U = 771.5, P = 0.42	-----------

Table 1.2. Pairwise test results of male size between the species used in the paper.

Species \ Species	Male size (mm) Turkey's pairwise				
	L.geometricus	*L. hasselti*	*L. mirabilis*	*L. mactans*	*L.hesperus*
L.geometricus	---------------	Q = 2.2, P = 0.54	Q = 1.1, P = 0.94	Q = 14.4, P < 0.001*	Q = 18.5, P < 0.001*
L. hasselti	Q = 2.2, P = 0.54	-----------	Q = 3.2, P = 0.16	Q = 12.1, P < 0.001*	Q = 15.8, P < 0.001*
L. mirabilis	Q = 1.1, P = 0.94	Q = 3.2, P = 0.16	------------	Q = 15.3, P < 0.001*	Q = 19.4, P < 0.001*
L. mactans	Q = 14.4, P < 0.001*	Q = 12.1, P < 0.001*	Q = 15.3, P < 0.001*	-----------	Q = 2.0, P = 0.62
L. hesperus	Q = 18.5, P < 0.001*	Q = 15.8, P < 0.001*	Q = 19.4, P < 0.001*	Q = 2.0, P = 0.62	-----------

Table 1.3. Pairwise test results of female mass between the species used in the paper.

Species \ Species	Female mass (mg)<br>Mann-Whitney pairwise				
	L.geometricus	*L. hasselti*	*L. mirabilis*	*L. mactans*	*L.hesperus*
L.geometricus	---------------	U = 922,<br>P = 0.99	U = 522,<br>P < 0.001*	U = 389,<br>P = 0.097	U = 463,<br>P < 0.001*
L. hasselti	U = 922,<br>P = 0.99	-----------	U = 508,<br>P < 0.001*	U = 365.5,<br>P = 0.14	U = 404.5,<br>P < 0.001*
L. mirabilis	U = 522,<br>P < 0.001*	U = 508,<br>P < 0.001*	------------	U = 170,<br>P < 0.001*	U = 202.5,<br>P < 0.001*
L. mactans	U = 389,<br>P = 0.097	U = 365.5,<br>P = 0.14	U = 170,<br>P < 0.001*	-----------	U = 326,<br>P = 0.032*
L. hesperus	U = 463,<br>P < 0.001*	U = 404.5,<br>P < 0.001*	U = 202.5,<br>P < 0.001*	U = 326,<br>P = 0.032*	-----------

Table 1.4. Pairwise test results of female size between the species used in the paper.

Species \ Species	Female size (mm)<br>Turkey's pairwise				
	L.geometricus	*L. hasselti*	*L. mirabilis*	*L. mactans*	*L.hesperus*
L.geometricus	---------------	Q = 2.4,<br>P = 0.42	Q = 4.6,<br>P = 0.013*	Q = 2.1,<br>P = 0.56	Q = 10.0,<br>P < 0.001*
L. hasselti	Q = 2.4,<br>P = 0.42	-----------	Q = 7.1,<br>P < 0.001*	Q = 0.2,<br>P = 0.99	Q = 7.6,<br>P < 0.001*
L. mirabilis	Q = 4.6,<br>P = 0.013*	Q = 7.1,<br>P < 0.001*	------------	Q = 6.6,<br>P < 0.001*	Q = 14.7,<br>P < 0.001*
L. mactans	Q = 2.1,<br>P = 0.56	Q = 0.2,<br>P = 0.99	Q = 6.6,<br>P < 0.001*	-----------	Q = 7.4,<br>P < 0.001*
L. hesperus	Q = 10.0,<br>P < 0.001*	Q = 7.6,<br>P < 0.001*	Q = 14.7,<br>P < 0.001*	Q = 7.4,<br>P < 0.001*	-----------

2. Statistical results of the timed behaviours analyzed during the progress of the mating trials.

Table 2.1. Pairwise test results of total male courtship behaviour (from the beginning of the trial until achieving the first copulation) between the species used in the paper.

Species / Species	Total male courtship duration<br>Turkey's pairwise				
	L.geometricus	*L. hasselti*	*L. mirabilis*	*L. mactans*	*L.hesperus*
L.geometricus	---------------	Q = 1.41, P = 0.85	Q = 3.33, P = 0.15	Q = 1.63, P = 0.78	Q = 0.53, P = 0.99
L. hasselti	Q = 1.41, P = 0.85	------------	Q = 5.27, P = 0.004*	Q = 3.35, P = 0.14	Q = 0.99, P = 0.97
L. mirabilis	Q = 3.33, P = 0.15	Q = 5.27, P = 0.004*	------------	Q = 5.27, P = 1.80	Q = 4.25, P = 0.032*
L. mactans	Q = 1.63, P = 0.78	Q = 3.35, P = 0.14	Q = 5.27, P = 1.80	---------	Q = 2.37, P = 0.46
L. hesperus	Q = 0.53, P = 0.99	Q = 0.99, P = 0.97	Q = 4.25, P = 0.032*	Q = 2.37, P = 0.46	-----------

Table 2.2. Pairwise test results of the pre-mounting male courtship (distal courtship, before mounting the female's ventral abdomen close to the epigine) between the species used in the paper.

Species / Species	Pre-mounting male courtship<br>Mann-Whitney pairwise				
	L.geometricus	*L. hasselti*	*L. mirabilis*	*L. mactans*	*L.hesperus*
L.geometricus	---------------	U = 9, P = 0.002*	U = 10, P = 0.009*	U = 22, P = 0.19	U = 9, P = 0.004*
L. hasselti	U = 9, P = 0.002*	-----------	U = 61, P = 0.83	U = 33.5, P = 0.102	U = 65 , P = 0.73
L. mirabilis	U = 10, P = 0.009*	U = 61, P = 0.83	-------------	U = 30 , P = 0.24	U = 42, P = 0.38
L. mactans	U =22, P = 0.19	U = 33.5, P = 0.102	U = 30, P = 0.24	-----------	U = 24.5 , P = 0.063
L. hesperus	U = 9, P = 0.004*	U = 65, P = 0.73	U = 42, P = 0.38	U = 24.5, P = 0.063	-----------

Table 2.3. Pairwise test results of post-mounting male courtship (proximal courtship, after mounting the female's venter and before achieving the first copulation) between the species used in the paper.

Species \ Species	Post-mounting male courtship Mann-Whitney pairwise				
	L.geometricus	*L. hasselti*	*L. mirabilis*	*L. mactans*	*L.hesperus*
L.geometricus	---------------	U = 42, P = 0.49	U = 2, P < 0.001*	U = 18, P = 0.092	U = 28, P = 0.20
L. hasselti	U = 42, P = 0.49	-----------	U = 3, P < 0.001*	U = 27.5, P = 0.042*	U = 44.5, P = 0.12
L. mirabilis	U = 2, P < 0.001*	U = 3, P <0.001*	------------	U = 6, P=0.0017*	U = 5, P < 0.001*
L. mactans	U = 18, P = 0.092	U = 27.5, P = 0.042*	U = 6, P = 0.0017*	-----------	U = 39.5 , P = 0.47
L. hesperus	U = 28 , P = 0.20	U = 44.5 , P = 0.12	U = 5 , P < 0.001*	U = 39.5, P = 0.47	-----------

Table 2.4. Pairwise test results of male number of alternating cycles of courting on the web and female's venter before achieving the first copulation between the species used in the paper.

Species \ Species	Male number of alternating cycles of courting on the web and female's venter before the first copulation Mann-Whitney pairwise				
	L.geometricus	*L. hasselti*	*L. mirabilis*	*L. mactans*	*L.hesperus*
L.geometricus	---------------	U = 19, P = 0.027*	U = 6, P = 0.0011*	U = 18, P = 0.092	U = 22, P = 0.12
L. hasselti	U = 19, P = 0.027*	-----------	U = 17, P = 0.0022*	U = 19, P = 0.014*	U = 29.5, P = 0.047*
L. mirabilis	U = 6, P = 0.0011*	U = 17, P =0.0022*	------------	U = 0, P < 0.001*	U = 0, P < 0.001*
L. mactans	U = 18 , P = 0.19	U = 19, P = 0.102	U = 0, P < 0.001*	-----------	U = 42.5, P = 0.87
L. hesperus	U = 22, P = 0.12	U = 29.5, P = 0.047*	U = 0, P < 0.001*	U = 42.5, P = 0.87	-----------

Table 2.5. Pairwise test results of the duration of the first copulation (palp insertion) between the species used in the paper.

Species / Species	First copulation duration<br>Turkey’s pairwise				
	L.geometricus	*L. hasselti*	*L. mirabilis*	*L. mactans*	*L.hesperus*
L.geometricus	---------------	Q = 1.38, P = 0.86	Q = 3.43, P = 0.13	Q = 2.50, P = 0.40	Q = 4.14, P = 0.040*
L. hasselti	Q = 1.38, P = 0.86	------------	Q = 5.27, P = 0.004*	Q = 1.30, P = 0.89	Q = 3.1, P = 0.20
L. mirabilis	Q = 3.43, P = 0.13	Q = 5.27, P = 0.004*	------------	Q = 6.29, P <0.001*	Q = 8.22, P <0.001*
L. mactans	Q = 2.50, P = 0.40	Q = 1.30, P = 0.89	Q = 6.29, P <0.001*	---------	Q = 1.64, P = 0.77
L. hesperus	Q = 4.14, P = 0.040*	Q = 3.1, P = 0.20	Q = 8.22, P <0.001*	Q = 1.64, P = 0.77	-----------
